# Determinants of stillbirth among reviewed perinatal deaths in Ethiopia

**DOI:** 10.3389/fped.2022.1030981

**Published:** 2022-11-28

**Authors:** Neamin Tesfay, Frehiwot Legesse, Mandefro Kebede, Fitsum Woldeyohannes

**Affiliations:** ^1^ Center of Public Emergency Management, Ethiopian Public Health Institutes, Addis Ababa, Ethiopia; ^2^ Health Financing Program, Clinton Health Access Initiative, Addis Ababa, Ethiopia

**Keywords:** stillbirth, perinatal death surveillance, delay three, congenital anomalies, Ethiopia

## Abstract

**Background:**

The global burden of stillbirth has declined over time. However, the problem is still prominent in South Asian and Sub-Saharan African countries. Ethiopia is one of the top stillbirth-reporting countries worldwide. Despite several measures taken to reduce the burden of stillbirth; the pace of decline was not as good as the post-neonatal death. Thus, this study is aimed at identifying potential factors related to stillbirth in Ethiopia based on nationally reviewed perinatal deaths

**Method:**

The national perinatal death surveillance data were used for this study. A total of 3,814 reviewed perinatal death were included in the study. Two model families,namely generalized estimating equation, and alternating logistic regression models from marginal model family were employed to investigate the risk factors of stillbirth. The alternating logistic regression model was selected as the best fit for the final analysis.

**Result:**

Among reviewed perinatal deaths nearly forty percent (37.4%) were stillbirths. The findings from the multivariate analysis demonstrated that the place of birth (in transit and at home), cause of death (infection, and congenital and chromosomal abnormalities), maternal health condition (women with complications of pregnancy, placenta, and cord), delay one (delay in deciding to seek care) and delay three (delay in receiving adequate care) were associated with an increased risk of having a stillbirth. On the other hand, maternal education (women with primary and above education level) and the type of health facility (women who were treated in secondary and tertiary health care) were associated with a decreased risk of having a stillbirth.

**Conclusion:**

The study identified that both individual (place of delivery, cause of death, maternal health condition, maternal education, and delay one) and facility level (type of health facility and delay three) factors contributed to stillbirth outcome. Therefore, policies that are aimed at encouraging institutional delivery, improving health seeking behavior, and strengthening facility-level readiness should be devised to reduce the high burden of stillbirth in Ethiopia.

## Introduction

Stillbirth (fetal death) is defined as the death of a fetus in the uterus before the onset of labor and during delivery (1). Stillbirth has significant national and individual implications; nationally, it is one of the important yardsticks used to assess the overall health system of a nation; and at an individual level, it can result in an unparalleled financial and psychological impact on parents (2). Globally, the rate of stillbirth has dropped from 21.4 per 1,000 live birth(LBs) in 2000, with a 2.3% annual rate of reduction(ARR), to 13.9 per 1,000 live birth(LBs) in 2019 (3). Despite the significant global reduction, the burden is still disproportionally high in Sub-Saharan African and South Asian countries (4).

Every Newborn Action Plan (ENAP) is one of the global initiatives designed to improve neonatal outcomes. In addition, strategies for ending preventable maternal mortality were also put in place to reduce inequality in service provision (5). To better frame those strategies, the world health organization (WHO) general assembly in 2014 adopted target of ENAP, which has a goal of reducing the stillbirth rate to only 12 deaths per 1,000 live birth by 2030 (6).

Ethiopia is one of the countries that has a very high burden of stillbirth (3). The country has reduced the rate of stillbirth from 35.8 per 1,000 LBs in 2000, with an ARR of 2.0%, to 24.6 per 1,000 LBs in 2019 (3, 7). However, compared to other health outcome indicators. the rate of reduction was rather slow and stagnant (8). The presence of noticeable regional variation is one of the reasons believed to have hampered the pace of decline at a national level (9).

One of the key strategies of ENAP is generating real-time information on every newborn death and facilitating decisions to improve the quality of service to avert similar preventable death in the future (10). To this effect, Ethiopia established the perinatal death surveillance and response system (PDSR) in 2017, which was integrated into the pre-existing maternal death surveillance and response (MDSR) system (11). Accordingly, in August 2017, the MDSR system was changed to a more comprehensive Maternal and Perinatal Death Surveillance and Response (MPDSR) system by harmonizing perinatal and maternal death surveillance systems (12).

MPDSR operates both at the community and health facility level and provides actionable data on perinatal death (mortality level, cause of death, contributing factors) in real time (13). The primary focus of PDSR system is to utilize the findings for planning appropriate and effective preventive actions; however, the system implementation has been challenged by low engagement of the community and poor utilization of the data to produce evidence-based decisions (14).

Ethiopia has adopted a multi-faceted approach to minimize the high burden of stillbirth and neonatal mortality. Beyond establishing MPDSR, some of the strategies employed include, enhancing behavioral and nutritional intervention, prevention and treatment of medical disorders, and Infection screening and monitoring during labour and delivery as well as Improving intrapartum care were the major ones (15–20). However, despite all this effort, Ethiopia is still facing a significantly high burden of stillbirth and the country has failed to achieve the national target set for 2020 (21). This is attributed to the lack of coordination, notable regional variation, and absence of a robust monitoring mechanism (22, 23).

Globally, 40% of stillbirths occur during labour, which signals the role of quality of intrapartum care in reducing the burden of stillbirth (24). In general, multiple factors could result in stillbirth, which can generally be categorized as maternal, fetal, and facility-level factors (25). Congenital malformation, infection, gestational age, sex, and birth weight are the fetal factors related to stillbirth (26–30), while maternal health conditions before and during pregnancy, maternal age, maternal parity, history of ANC visit, mode of delivery, maternal education, wealth index, birth interval, previous history of stillbirth, fetal movement monitoring are the major maternal factors (31–35). On the other hand, factors such as intrapartum monitoring, clinical management, and capacity of diagnosis are some of the facility-level predictors of stillbirth (36).

Although Ethiopia has a very high burden of stillbirth, the area has received limited attention when it comes to generating scientific evidence. Therefore, this study—among others—is aimed at identifying risk factors that are associated with stillbirth by considering both individual and facility-level factors.

## Methods

### Study setting

Ethiopia has an estimated population of 117,876,000 in 2021, out of which 17, 216,372 are under-five children (37). Administratively, Ethiopia has nine regions and two city administrations, namely Tigray, Afar, Amhara, Oromia, Somali, Benishangul-Gumuz, Southern Nations Nationalities, and Peoples Region (SNNPR), Gambella, Harari, Addis Ababa city administration and Dire Dawa city administration (38). The country has high infant, under-five, and maternal mortality (47 per 1000LBs), (59 per 1000LBs), and (412 per 100,00 LBs), respectively (39, 40).

### Data source and study participant

The study used data from Ethiopian Public Health Institutes (EPHI); perinatal death data obtained from all PDSR implementing regions for four consecutive years (2018–2021) were utilized for the study. The source population of the study are all perinates who died and were reviewed by the MPDSR committee during the study period. Accordingly, a total of 3,814 reviewed perinatal deaths were included in the study. The PDSR data was hierarchical i.e., perinates were nested in 161 reporting health facilities and 45 provinces of the country.

### Study variables

#### Outcome variable

The dependent variable of this study is the outcome of the fetus after birth, which is classified as “Stillbirth” and “live birth”.

#### Explanatory variables

Both individual and facility-level variables were included as a predictor in the model. Sex, gestational age, place of birth, mode of delivery, and assigned cause of death were included as neonatal factors in the model. The medical cause of death was incorporated as individual death after the underlying cause of death was assigned using the International Classification of Diseases -Perinatal Mortality (ICD-PM) (41). On the other hand, maternal factors variables such as maternal age, maternal parity, educational status, number of antenatal care (ANC) visits, the score of delay one, and maternal health condition were included in the model. Moreover, maternal health conditions were assigned per the guidance of ICD-PM. The score of delay one, which is a delay in deciding to seek care (42), was computed using the row sum of seven variables included under this domain; namely (1) family poverty, (2) bad experience with previous health care, (3) failure to recognize the danger signs of pregnancy, (4) lack of awareness on where to seek care, (5) lack of person to take care of other children, (6) reliant on traditional practice and (7) lack of decision to go to a health facility. All of them were binary variables with “Yes” and “No” responses; and after summation of the score, to keep the normality of the data a square root transformation was carried out (43). Finally, the transformed variable was treated as continuous variables to make a parsimonious model (44).

At a facility (community) level; variables such as residence, type of region, type of health facility, a score of delay two, and a score of delay three were taken into consideration. The type of region was classified into three categories (city, agrarian, and pastoralist) based on the cultural and socio-economic backgrounds of the population (45). Furthermore, the type of facility was codified into classes (primary, secondary, and tertiary facilities) according to their manpower, medical equipment, and service provision (46). Moreover, the score of delay two(a delay to reach the facility that provides emergency obstetric care (EmONC) and delay three(a delay that occurs in receiving care after arrival at the health facility) (42), were computed similarly to the score of delay one. The score of delay two was computed using four questions: namely (1) absence of transportation, (2) expensive cost of transportation, (3) no facility within a reasonable distance and (4) poor road condition. Similarly, the score of delay three was also computed using four questions; namely, (1) long travel time from health facility to health facility (due to multiple referrals to receive optimal care), (2) long waiting time before receiving treatment, (3) mistake during an assessment, diagnosis, and treatment and (4) shortage of equipment and supplies. Both delays (two and three) were measured using binary variables and the responses were set as “Yes” and “No” options.

### Case definition

#### Case definition (extended perinatal death)

Death of a fetus born after 28 completed weeks of gestation or neonatal deaths through the first 28 completed days after birth (11).

#### Stillbirth

Stillbirth is a fetal death (i.e., death before the complete expulsion or extraction of a product of conception from its mother) in the third trimester (≥28 completed weeks of gestation) or with birth weight ≥1,000 g or crown to heel length ≥35 cm (22).

#### Operational definition

Perinatal deaths were categorized by the time of death; antepartum, intrapartum stillbirth, unknown and neonatal. Cause of death during the antepartum and intrapartum, was taken as the cause of stillbirth in the study. Furthermore, the contributing maternal conditions were classified into five major categories per the guidance of ICD_PM_10 (Table 1) (47).

**Table 1:** ICD-PM categories with the specific cause of perinatal death and maternal health condition.

Status during birth	Time of death	Category	Description	Example
Stillbirth	Antepartum death	A1	Congenital malformations and chromosomal abnormalities	Anencephaly, encephalocele, microcephaly, congenital hydrocephalus, spina bifida, etc.
		A2	Infection	Congenital syphilis, congenital malaria, congenital rubella syndrome, congenital TB, etc.
		A3	Antepartum hypoxia	Intrauterine hypoxia
		A4	Other specified antepartum disorder	Vasa previa, ruptured cord, twin-twin transfusion, Intraventricular (nontraumatic) haemorrhage, rhesus and ABO isoimmunization, etc.
		A5	Disorders related to fetal growth	Small for gestational age, macrosomia, post-term, etc.
		A6	Antepartum death of unspecified cause	Intrauterine death of unspecified cause
	Intrapartum death	I1	Congenital malformations and chromosomal abnormalities	Anencephaly, encephalocele, microcephaly, congenital hydrocephalus, spina bifida, etc.
		I2	Birth trauma	Intracranial laceration and haemorrhage due to birth injury, fracture of the skull due to birth injury, etc.
		I3	Acute intrapartum event	Intrauterine hypoxia
		I4	Infection	Congenital syphilis, congenital malaria, congenital rubella syndrome, congenital TB, etc.
		I5	Other specified intrapartum disorder	Vasa previa, ruptured cord, twin-twin transfusion, intraventricular (nontraumatic) haemorrhage, rhesus and ABO isoimmunization, etc.
		I6	Disorders related to fetal growth	Small for gestational age, extremely low birth weight, macrosomia, post-term, etc.
		I7	Intrapartum death of unspecified cause	Fetal death of unspecified cause
Live birth	Neonatal death	N1	Congenital malformations, and chromosomal abnormalities	Anencephaly, encephalocele, microcephaly, congenital hydrocephalus, spina bifida, etc.
		N2	Disorders related to fetal growth	Small for gestational age, exceptionally large baby, post-term, etc.
		N3	Birth trauma	Cerebral haemorrhage due to birth injury, intraventricular haemorrhage due to birth injury, etc.
		N4	Complications of intrapartum events	Intrauterine hypoxia, birth asphyxia
		N5	Convulsions and disorders of cerebral status	Neonatal cerebral irritability, neonatal cerebral depression, neonatal coma, etc.
		N6	Infection	Tetanus neonatorum, bacterial meningitis, bacterial sepsis, congenital pneumonia, etc.
		N7	Respiratory and cardiovascular disorders	Respiratory distress syndrome, neonatal aspiration syndromes, neonatal cardiac failure, neonatal cardiac dysrhythmia, neonatal hypertension, etc.
		N8	Other neonatal conditions	Vasa previa, ruptured cord, twin-twin transfusion, rhesus and ABO isoimmunization, kernicterus, etc.
		N9	Low birth weight and prematurity	Extremely low birth weight, extreme immaturity
		N10	Miscellaneous	Cases where codes from several other sections of ICD-10 should be used
		N11	Neonatal death of unspecified cause	Congenital renal failure, termination of pregnancy, affecting fetus and newborn, withdrawal symptoms from drug
Maternal conditions		M1	Complications of the placenta, cord and membranes	Abruptio placentae, prolapsed cord, chorioamnionitis, etc.
		M2	Maternal complications of pregnancy	Premature rupture of membranes (PROM), oligo- and polyhydramnios, ectopic pregnancy, multiple pregnancy, etc.
		M3	Other complications of labour and delivery	Breech delivery and extraction, forceps delivery, caesarean delivery
		M4	Maternal medical and surgical conditions	hypertensive disorders, maternal injury, maternal use of tobacco, alcohol or drugs, etc.
		M5	No maternal conditions	No condition identified

### Data management and statical analysis

The data was exported from Epi -info version 7.2 to Stata version 17 for data cleaning and further analysis. Using the cleaned data descriptive [count, mean, percentage, and standard deviation(SD)] and analytical analysis (marginal model) was carried out and reported.

#### Model building

Both bi-variable and multivariable analyses were performed to determine the existing association with the outcome of interest. Initially, bivariate analysis was performed and variables with a *p*-value of 0.2 and below were retained in the model. Secondly, multicollinearity was assessed using variance inflation factor (VIF) before proceeding to multivariate analysis.

#### Marginal model

Generalized Estimating Equation (GEE) model is used to describe changes in the population while accounting for the within-cluster correlation of observations (48–50). The marginal models fitted in this study are GEE and Alternating Logistic Regression (ALR). Both marginal models were used to model the birth outcome and compare the odds of stillbirth outcome by considering the respective risk factors. Within the GEE and ALR model frameworks, health facilities were clustered, and not independent within each health facility. The response variables were the occurrence of stillbirth (Yi: binominal variable, i.e., stillbirth or live birth); and for this study, we considered that the event occurred if the perinate died before and during the onset of labour. Consequently, the response variable was coded with 1 for birth outcome with stillbirth and 0 for the alive birth outcome. The link function between the mean value $Y_{i}$ and the model covariates considered for GEE, are defined by the ` working correlation structure stated below:

$$g(\pi_{j})=\mathrm{logit}\;(\pi_{\;j})=x_{j}\beta$$

Where $g(\pi_{\;j})$ is logit link function, $x_{i}$ is $n_{i}*x_{i}$ -dimensional vector of known covariates, β=(1xp) dimensional vector of unknown parameters, $E(Y_{i})=\pi_{i}$ is the expected value of the response $Y_{i}$ in the cluster *i* which is binomially distributed as $y_{i}∼\mathrm{bin}(n_{i},\pi_{i})$. In addition, GEE is the non-likelihood method that captures the association within health facilities in terms of marginal correlation (50). With this GEE model, the correlation structure of the data within each health facility was assumed to be independent, unstructured, exchangeable, and first-order auto-regressive(51). Furthermore, Independence and exchangeable working assumptions can be used in virtually all applications, whether longitudinal, clustered, multivariate, or otherwise correlated. However, autoregressive AR (1) and unstructured correlation structures are less relevant for clustered data (50). In general, the parameter *β* is estimated by quasi-likelihood. However, when the cluster sizes become larger, the simultaneous estimation of marginal mean and dependence structure can become computationally prohibitive using GEE. As a result, alternating logistic regression measures the pairwise association of two observations in the same health facility and follows the precision estimates for the regression (*β*) and the association (*α*) parameters considered (50, 52). Furthermore, unlike GEE, no working assumptions about the thirdorder and fourth-order odds ratios are required. Alternating logistic regression measures the association using the odds ratio, which is interpretable and more applicable for binary data (50, 52). Let $y_{ijk}$ be the log odds ratio between outcomes $y_{ij}$ and $y_{ik}$, let $u_{ij}=p(y_{ij}=1)$ and $v_{ijk}=p(y_{ij}=1,\;y_{ik}=1)$, then the association of the two responses is defined as (53):

$$logit_{p}(Y_{ij}=1/Y_{ik}=y_{ik})=y_{ikj}y_{ik}+\log\left(\frac{\mu_{ij\;}-v_{ijk}}{1-\;u_{ij}-u_{ik}+\;u_{ijk}}\right)$$

Similar to GEE, the parameter *β* is estimated by quasi-likelihood.

The descriptive statics (count, mean, percentage, and Standard deviation) were computed using the Stata version 17, while GEE and ALR models fit for this study were carried out with the SAS 9.4 version.

#### Model selection

The final model was built based on the principle of generality, the goodness of fit, and parsimony. In this study, exchangeable and independence were the two working correlation assumptions of the GEE model. The best-fitted model was selected based on the minimum quasi-information criteria (QIC) and standard error, where the two criteria were used to select the working correlation structure (54). The backward selection strategy was adopted for the selection of the final variables related to stillbirth. With this method, we started a full model containing all main effects and interactions and continued the process until we get a parsimonious model. After obtaining the best-fitted model through GEE, we used the ALR model to decide the final variables fitted to the model. Furthermore, during ALR estimation, the logs odds ratio model was specified using SAS software's nesting function “NEST1”. Subsequently, we specified the function using “SUB_CLUST = Health facility” to define the subcluster(Health facilities) within the cluster(Provinces) (55). Lastly, the odds ratio of the final model was computed using the following formula $OR=e^{\beta }$ (56).

## Result

### Selected characteristics of reported facilities

A total of 3,814 perinatal deaths were included in the study, out of which 1,417(37.2%) were stillbirths. Among reporting health facilities, nearly half (49.6%) of the reported perinatal deaths from primary level health care providers were stillbirths. Region-wise, 56.5%,47.7%, and 44.4% of the reported perinatal deaths from Oromia, Amahara, and Benshangul Gumuz were stillbirths. Furthermore, 42.5% of the reported perinatal deaths in 2020 were stillbirths (Table 2).

**Table 2:** Selected background characteristics of reporting facilities by outcome status of the perinate in Ethiopia,2021.

Characteristic	Overall, *N* = 3814	Alive birth, *N* = 2397	Stillbirth, *N* = 1417
**Level of care**			
Primary level health care	1,999	1,008 (50.4%)	991 (49.6%)
Secondary level health care	878	515 (58.7%)	363 (41.3%)
Tertiary level health care	937	874 (93.3%)	63 (6.7%)
**Facility Ownership**			
Government	3,801	2,391 (62.9%)	1,410 (37.1%)
NGO	2	2 (100.0%)	0 (0.0%)
Private	11	4 (36.4%)	7 (63.6%)
**Source of data**			
FBAF	3,639	2,323 (63.8%)	1,316 (36.2%)
VA	175	74 (42.3%)	101 (57.7%)
**Reporting region**			
Addis Ababa	808	761 (94.2%)	47 (5.8%)
Amhara	1,989	1,041 (52.3%)	948 (47.7%)
Benishangul-Gumuz	72	40 (55.6%)	32 (44.4%)
Dire Dawa	38	30 (78.9%)	8 (21.1%)
Gambella	4	4 (100.0%)	0 (0.0%)
Harer	21	21 (100.0%)	0 (0.0%)
Oromia	568	247 (43.5%)	321 (56.5%)
Sidama	96	84 (87.5%)	12 (12.5%)
SNNP	154	114 (74.0%)	40 (26.0%)
Somali	64	55 (85.9%)	9 (14.1%)
**Year of reporting**			
2018	448	346 (77.2%)	102 (22.8%)
2019	782	452 (57.8%)	330 (42.2%)
2020	879	505 (57.5%)	374 (42.5%)
2021	1705	1,094 (64.2%)	611 (35.8%)

### Selected characteristics of the mothers of the deceased perinates

The average age of the mother with a stillbirth outcome was 27.4 with a standard deviation (SD) of 5.70 years. Similarly, the average maternal parity among mothers who encountered stillbirth outcomes was 2.6 with an SD of 1.88. The proportion of stillbirths was higher among women who are uneducated (46.8%) as compared to women who attended primary education (23.5%). Likewise, mothers with maternal complications of pregnancy had a higher proportion of stillbirth (66.6%) as compared to mothers who had no identified maternal complication (30.2%). Furthermore, mothers who live in rural areas had a higher proportion of stillbirths (46.7%) than mothers who lived in urban areas (25.6%) (Table 3).

**Table 3:** Selected background characteristics of the deceased perinate's mother by outcome status of the perinate in Ethiopia, 2021.

Characteristic	Overall, *N* = 3,814	Alive birth, *N* = 2,397	Stillbirth, *N* = 1,417
**Maternal age** ^a^	27.3 (5.37)	27.3 (5.16)	27.4 (5.70)
**Number of ANC visit** ^a^	2.6 (1.47)	2.7 (1.45)	2.3 (1.47)
**Maternal parity** ^a^	2.4 (1.73)	2.3 (1.63)	2.6 (1.88)
**Religion**			
Christian	2,978	1,829 (61.4%)	1,149 (38.6%)
Muslim	812	550 (67.7%)	262 (32.3%)
Traditional	24	18 (75.0%)	6 (25.0%)
**Maternal education**			
Uneducated	2,168	1,153 (53.2%)	1,015 (46.8%)
Primary	941	720 (76.5%)	221 (23.5%)
Secondary and above	705	524 (74.3%)	181 (25.7%)
**Maternal health condition**			
Other complications of labour and delivery	140	67 (47.9%)	73 (52.1%)
Maternal medical and surgical conditions	295	199 (67.5%)	96 (32.5%)
Maternal complications of pregnancy	326	109 (33.4%)	217 (66.6%)
Complications of the placenta, cord, and membranes	389	163 (41.9%)	226 (58.1%)
No maternal conditions identified	2,664	1,859 (69.8%)	805 (30.2%)
**Status of the mother**			
Alive	3,353	2,241 (66.8%)	1,112 (33.2%)
Died	461	156 (33.8%)	305 (66.2%)
**Type of region**			
Pastoralist	140	91 (70.7%)	41 (29.3%)
City administration	812	812 (93.7%)	55 (6.3%)
Agrarian	1486	1,486 (52.9%)	1,321 (43.1%)
**Residence**			
Rural	2098	1,118 (53.3%)	980 (46.7%)
Urban	1716	1,279 (74.5%)	437 (25.6%)

^a^ Mean (SD); *n* (%).

### Selected characteristics of the deceased perinate

The average gestational week of stillbirth was 36.0 with an SD of 3.03. The proportion of stillbirth was higher among perinates who were delivered by spontaneous vaginal delivery (40.0%) as compared to perinates delivered by caesarean section (20.4%). Besides, perinates who were born in transit had a high proportion of stillbirths (71.8%) as compared to perinates who were born in a health facility (35.1%) (Table 4).

**Table 4:** Selected characteristics of the perinate by outcome status in Ethiopia, 2021.

Characteristic	Overall, *N* = 3,814^a^	Alive birth, *N* = 2,397^a^	Stillbirth, *N* = 1,417^a^
**Estimated gestational age** ^a^	35.5 (3.45)	35.3 (3.65)	36.0 (3.03)
**Sex**			
Male	2200	1,418 (64.5%)	782 (35.5%)
Female	1614	972 (60.7%)	635 (29.3%)
**Place of death**			
Health facility	3,348	2,293 (68.5%)	1,055 (31.5%)
Home	354	75 (21.2%)	279 (78.8%)
On Transit	112	29 (25.9%)	83 (74.1%)
**Place of birth**			
Health facility	3,478	2,258 (64.9%)	1,220 (35.1%)
Home	265	119 (44.9%)	146 (55.1%)
In transit	71	20 (28.2%)	51 (71.8%)
**Mode of delivery**			
Cesarean section	520	414 (79.6%)	106 (20.4%)
Operative vaginal delivery	223	139 (62.3%)	84 (37.7%)
Spontaneous vaginal delivery	3,071	1,844 (60.0%)	1,227 (40.0%)

^a^ Mean (SD); *n* (%).

### Assigned cause of death to the deceased perinate

The cross-cutting causes of death (birth trauma (44.4%), infection (56.3%), and congenital malformations, deformations, and chromosomal abnormalities (51.3%)) for both live and stillbirth had a relatively similar proportion of contribution to stillbirth (Table 5).

**Table 5:** Assigned cause of death by the outcome of the perinate in Ethiopia, 2021.

Characteristic	Overall, *N* = 3,814^a^	Alive birth, *N* = 2,397^a^	Stillbirth, *N* = 1,417^a^
**Assigned Cause of death**			
Antepartum death of unspecified cause	1	0 (0.0%)	1 (100.0%)
Other specified antepartum disorder	2	0 (0.0%)	2 (100.0%)
Intrapartum death of unspecified cause	2	0 (0.0%)	2 (100.0%)
Convulsions and disorders of cerebral status	4	4 (100.0%)	0 (0.0%)
Birth trauma	18	10 (55.6%)	8 (44.4%)
Acute antepartum event	25	0 (0.0%)	25 (100.0%)
Other neonatal conditions	25	25 (100.0%)	0 (0.0%)
Respiratory and cardiovascular disorders	110	110 (99.1%)	0 (0.0%)
Congenital malformations, deformations, and chromosomal abnormalities	267	130 (48.7%)	137 (51.3%)
Disorders related to fetal growth	274	0 (0.0%)	274 (100.0%)
Acute intrapartum event	339	0 (0.0%)	339 (100.0%)
Complications of intrapartum events	682	682 (100.0%)	0 (0.0%)
Low birth weight and prematurity	946	946 (100.0%)	0 (0.0%)
Infection	1119	492 (44.0%)	627 (56.0%)

^a^ Stands for mean and standard deviation.

### Delay factor for perinatal death

The proportion of stillbirth due to delay one was higher among perinates whose mothers were delayed in seeking care due to lack of decision to go to a health facility (54.2%) than those who were delayed in seeking care due to previous bad experiences in health facilities (18.2%). Similarly, the proportion of stillbirth because of delay two was higher among perinates whose mothers were delayed in reaching care due to expensive cost of transportation (62.3%) as compared to those who were delayed reaching care due to the absence of health facilities with reasonable distance (42.1%). Moreover, the proportion of stillbirths because of delay three was higher among perinate mothers who were not assessed and treated properly (63.9%) than those who travelled for a longer duration to get into the referred facility (33.3%) (Table 6).

**Table 6:** Delay factors contribute to perinatal death by outcome status of the perinate in Ethiopia, 2021.

Characteristic	Overall, *N* = 3,814^a^	Alive birth, *N* = 2,397^a^	Stillbirth, *N* = 1,417^a^
**Delay 1—Decision to seek care**			
Family poverty	257	183 (71.2%)	74 (28.8%)
Bad experience with previous healthcare	11	9 (81.8%)	2 (18.2%)
Failed to recognize the danger of pregnancy	1104	603 (54.6%)	501 (45.4%)
Unaware of where to go	54	28 (51.9%)	26 (48.1%)
Absence of child care	50	30 (60.0%)	20 (40.0%)
Reliant on traditional practice	37	23 (62.2%)	14 (37.8%)
Lack of decision to a health facility	454	208 (45.8%)	246 (54.2%)
**Delay 2—Reaching care**			
Absence of transportation	227	130 (57.3%)	97 (42.7%)
Expensive cost of transportation	53	20 (37.7%)	33 (62.3%)
No facility within a reasonable distance	126	73 (57.9%)	53 (42.1%)
Poor road condition	103	51 (49.5%)	52 (50.5%)
**Delay 3—Receiving care**			
Long travel time from health facility to health facility	804	536 (66.7%)	268 (33.3%)
Long waiting time before treatment was received	521	228 (43.8%)	293 (56.2%)
Mistaken during the assessment, diagnosis, and treatment	205	74 (36.1%)	131 (63.9%)
Shortage of equipment and supplies	482	178 (36.9%)	304 (63.1%)

^a^ *n* (%).

### Marginal model

The marginal model was used to determine the factors that affect early neonatal death. In the analysis, both individual and facility-level factors were considered in the model.

#### Predictor of stillbirth

The model with the smallest QIC and standard error was selected as the best-fitted model in the study. Accordingly, the ALR model was selected as the best-fitted model. Per the ALR model, place of birth, cause of death (infection and congenital and chromosomal abnormalities), maternal health condition, maternal education status, delay one, type of health facility, and delay three were predictors of stillbirth.

The analysis output under ALR model suggests that the place of birth had a significant association with stillbirth. It was revealed that perinate who were delivered in transit had exp(0.86) = 2.36 times higher odds of stillbirth outcome than those who were delivered at a health facility. Perinates who were delivered at home had exp(0.32) = 1.38 times higher odds of stillbirth outcome than those delivered at a health facility.

In addition, a statistically significant relationship between infection and stillbirth was demonstrated. The odds of giving a stillbirth were much higher among perinates with infection [exp(0.77) = 2.16] than perinates without infection. Similarly, perinates with congenital malformations, deformations, and chromosomal abnormalities had a higher risk of stillbirth [exp(1.07) = 2.92] as compared to perinate without congenital and chromosomal abnormalities.

With respect to the maternal health condition, it was indicated that a mother with placenta, cord, and membranes complications, and a mother with maternal complications of pregnancy, were significantly related to stillbirth. This indicates that the estimated odds of having a stillbirth in women who had placenta, cord, and membranes complications were exp.(0.26) = 1.30 times higher than a woman without any of those maternal complications. Similarly, the odds of having a stillbirth in women who had complications of pregnancy was exp.(0.78) = 2.18 times higher compared to women without any maternal complications.

Maternal education also has a role in the stillbirth outcome, as indicated in Table 2, women with a secondary and above level of education were associated with exp(−0.25) = 0.78 times fewer odds of having stillbirth compared to women with no education. Moreover, women with primary level education had exp(−0.43) = 0.65 times less risk of having stillbirth compared to their counterparts with no education. In line with this, as the score of delay-one increases by one unit, the odds of having a stillbirth increase exp(0.37) = 1.45 times.

As depicted in Table 3, the type of healthcare provider is significantly related to stillbirth. The estimated odds of having stillbirth among women who were treated in secondary health care was exp.(−0.91) = 0.40 times lower than the estimated odds for women who were treated in primary health care. Likewise, women who were treated in tertiary health care had exp(−1.82) = 0.16 times less risk of having stillbirth compared to their counterparts treated in primary health care providers. Furthermore, as a score of delay three increases by one unit, the odds of having a stillbirth increased by exp(0.80) = 2.23 times (Table 7).

**Table 7:** Parameter estimates and empirical standard errors of GEE and ALR models for stillbirth.

Marginal		Exchangeable		Independent		ALR	
Effect	Par	Est (SE)	*p*-value	Est (SE)	*p*-value	Est (SE)	*p*-value
**Intercept**	β0	−0.88 (0.14)	<0.0001	−1.10 (0.10)	<0.0001	−1.00 (0.23)	<0.0001
**Place of birth (ref = Health facility)**							
On transit	β1	0.97 (0.27)	0.0003	1.26 (0.31)	<0.0001	0.86 (0.24)	0.0004
Home	β2	0.34 (0.15)	0.0215	0.47 (0.15)	0.0021	0.32 (0.14)	0.0185
**Infection (ref = no)**							
Yes	β3	0.91 (0.08)	<0.0001	1.23 (0.09)	<0.0001	0.77 (0.10)	<0.0001
**Congenital malformations, deformations and chromosomal abnormalities (ref = no)**							
Yes	β4	1.10 (0.15)	<0.0001	1.30 (0.16)	<0.0001	1.07 (0.15)	<0.0001
**Maternal health condition (ref = No maternal conditions)**							
Other complications of labor and delivery	β5	0.19 (0.18)	0.3083	0.38 (0.19)	0.0469	0.17 (0.17)	0.3352
Maternal medical and surgical condition	β6	0.02 (0.15)	0.9025	0.07 (0.16)	0.6675	0.06 (0.14)	0.6909
Maternal complications of pregnancy	β7	0.88 (0.13)	<0.0001	1.22 (0.14)	<0.0001	0.78 (0.13)	<0.0001
Complications of placenta, cord, and membranes	β8	0.30 (0.12)	0.0151	0.62 (0.13)	<0.0001	0.26 (0.12)	0.0301
**Maternal level of education (ref = no education)**							
Secondary and above	β9	−0.23 (0.11)	0.0304	−0.57 (0.11)	<0.0001	−0.25 (0.10)	0.0139
Primary	β10	−0.48 (0.10)	<0.0001	−0.64 (0.11)	<0.0001	−0.43 (0.10)	<0.0001
**Score of delay one**	β11	0.34 (0.08)	<0.0001	0.46 (0.07)	<0.0001	0.37 (0.08)	<0.0001
**Type of region (ref = agrarian)**							
Pastoralist	β12	−0.72 (0.38)	0.061	−0.63 (0.22)	0.0033	−0.38 (0.57)	0.5084
City	β13	−1.03 (0.36)	0.0039	−1.14 (0.22)	<0.0001	−0.42 (0.46)	0.3542
**Level of care (ref = Primary)**							
Secondary LHC	β14	−0.48 (0.25)	0.057	−0.03 (0.10)	0.7857	−0.91 (0.27)	0.0007
Tertiary LHC	β15	−1.54 (0.38)	<0.0001	−1.44 (0.21)	<0.0001	−1.82 (0.40)	<0.0001
**Score of delay three**	β16	0.81 (0.08)	<0.0001	0.59 (0.07)	<0.0001	0.80 (0.09)	<0.0001
**Alpha1 (Within subcluster)**						1.42 (0.20)	<0.0001
**Alpha2 (between subcluster)**						0.74 (0.36)	0.0409
**QIC**		4307.8523		4285.8152		4209.9376	

## Discussion

This study utilized national PDSR data to identify the risk factors associated with stillbirth among reviewed perinatal deaths. The ALR analysis revealed that covariates like place of birth, cause of death (infection, congenital and chromosomal abnormalities), maternal health condition, maternal education status, delay one, type of health facility, and delay three were significantly associated with stillbirth.

The statistical analysis output revealed that place of birth is one of the important factors associated with stillbirth. The risk of having stillbirth was higher among perinates delivered in transit and at home as compared to those delivered at a health facility. This finding corresponds well with studies conducted in Bangladesh (57), and other five countries (India, Guatemala, Kenya, Pakistan, and Zambia) (58). This could be due to the fact that institutional delivery paves the way to early identification and management of intrapartum complications including the provision of cesarian section service, which has a significant role in the reduction of intrapartum stillbirth (59). In Ethiopia, only 48% and 43% of the women give birth at a health facility and had more than 4 ANC visits, respectively (39). However, ANC visits, levels of education, residence, and previous history of home delivery were major factors that influence institutional deliveries (60, 61). In general, the finding indicates that improving the coverage of institutional delivery is needed to reduce the high burden of stillbirth in the country.

The study also revealed that stillbirth due to infection was much higher compared to other causes. The finding was coherent with studies conducted in Ethiopia (Bahardar, Tigray, and Harir) (62–64), Afghanistan (65), Bangladesh (66), and Italy (67). The potential reasons that could explain those differences are (1) the presence of maternal infection which might lead to systemic illness in the mother, causing fetal death due to high maternal fever, respiratory distress, or other systemic reactions, without an infection being transmitted to the placenta or fetus (while the fetus is intact). (2) the second possible reason could be related to the infection of the placenta which results in reduced fetoplacental blood flow. Syphilis, malaria, and chorioamnionitis are the commonest causes of infection-related stillbirth in underdeveloped nations (68, 69). Considering this, putting in place effective measures such as improving the screening and treatment of syphilis and malaria should be coupled with interventions focused on preterm and term PROM to reduce the burden of stillbirth (70).

The study has also revealed that congenital malformations, deformations, and chromosomal abnormalities were associated with stillbirth outcomes, i.e., there is a threefold higher risk of stillbirth among perinate with congenital abnormalities than their counterparts. This finding was consistent with studies conducted in Ethiopia (71), Cameroon (28), and India (26). This could be due to a lack of maturity of the vital organs for fetal survival making the fetus prone to fatal complications and death. Neural tube defects, orofacial clefts, masculo-skeletal systemdefects, syndrome disorders, and cardiovascular system problems were prevalent congenital anomalies in Ethiopia (72).ANC visits and residence are known to have a significant role in the formation of congenital and chromosomal anomalies in Ethiopia (73). However, the lack of effective national surveillance, which can monitor and guide the prevention initiative, has untrimmed the efforts to the reduction of stillbirths due to congenital abnormalities in Ethiopia.

Women with maternal complications of pregnancy and complications of the placenta, cord, and membranes had a higher risk of having stillbirth as compared to women with no identified complication. The finding was complementary to studies conducted in Ethiopia (74), Iran (75), India (76), and Pakistan (77). This might be explained by the deteriorated maternal condition resulting in vessel compression and cessation of blood flow, as well as fetal perfusion. lastly, the combination of all these effects could lead to fetal asphyxia and death (78, 79). In line with this, history of ANC visits, urinary tract infection, being anemic, previous history of cesarean section, gestational hypertension, and diabetes were known modifiable risk factors for both maternal health conditions (i.e., Maternal complication of pregnancy and complications of the placenta, cord, and membranes) (30, 80, 81). Thus, the reduction of preventable stillbirths from the above two maternal health conditions can be achieved by controlling the predisposing factors.

Maternal education is the other variable that Is strongly associated with stillbirth outcomes. Educated women had a lower risk of having stillbirth as compared to uneducated women. In addition, the study revealed that the score of delay one had a positive dose-response relationship with stillbirth. This finding was congruent with studies conducted in Ethiopia (62), Nigeria (82), Nepal (35), Japan (83), and other six countries(Argentina, the Democratic Republic of the Congo, Guatemala, India, Pakistan, and Zambia) (84). This could be due to the assumption that educated women would have better knowledge, which enables them to make an early decision regarding their health. Considering the benefit of education, Ethiopia has launched a health extension program, which is designed to provide health education and basic health service at the community level (85). On top of this, Community Based Newborn Care (CBNC) and Integrated Community Case Management (iCCM) were included to foster the program (86). However, its implementation has been challenged due to health system (lack of regular monitoring, lack of logistics, and absence of continuous professional development activities) and community level (limited engagement of the community structure) challenges (87, 88). Overall, the finding indicated that health extension programs should be revitalized and coupled with other programs that could enhance the health-seeking behavior of women.

The type of health facility and the score of delay three were the other variables that are associated with stillbirth outcomes. Women who were treated in secondary and tertiary health care had a lower risk of having stillbirths as compared to women who were treated in primary health care. Besides, a positive dose-response relation was observed between a score of delay three and stillbirth outcomes. The finding was parallel with studies conducted in Nigeria (89), Jordan (90), Brazil (91), and Sweden (92). This might be explained by the level of health service providers' capacity of identifying and managing obstetrics emergencies. As the diagnostic capacity of the health facility increases, the risk of having an adverse outcome in fetal and maternal health will be reduced. Furthermore, the finding was concurrent with the evidence generated by the Ethiopian Service and Availability Readiness Assessment (SARA) report, where secondary and tertiary health facilities were proven to have better service availability and readiness in the provision of Emergency Newborn care (EmNeC) services (93). In addition to this, the country has invested a lot of resources in the establishment and expansion of neonatal intensive care units (NICUs) at secondary and tertiary levels of care to improve neonatal outcomes (94). Considering all this, the finding implies the presence of gaps in managing obstetrics emergencies at the lower level, in addition to poor referral linkage among health facilities.

The study has the following limitation that needs to be acknowledged. (1) All identified, confirmed, and reported perinatal death through a weekly reporting system were not reviewed and sent through the Perinatal Death Reporting Format (PDRF) to the next level, which might introduce potential bias to the study. (2) nearly all deaths were reported and reviewed from public facilities with limited involvement of private health facilities, and this could affect the representativeness of the study. (3) Lack of national guidelines and capacity for investigation of perinatal death, placental examination, genetic evaluation, and radiologic evaluation influenced the study in obtaining more in-depth data.

## Conclusion

Almost four in ten perinatal deaths were stillbirths. Place of delivery, cause of death, maternal health condition, maternal education status, delay one, type of health care, and delay three were predictors of stillbirth. Tailored intervention should be provided to the two major causes of stillbirth (infection and congenital malformations, deformations, and chromosomal abnormalities) by improving prenatal care and ANC visit. Moreover, intervention should focus on encouraging institutional delivery by improving the health-seeking behaviour of the community. One potential solution to this could be the revitalization of the existing health extension program. Furthermore, improving the managing capacity of obstetrics emergencies at a lower level should be enhanced in terms of manpower and equipment.

## Data Availability

The raw data supporting the conclusions of this article will be made available by the authors per the guidance of the data sharing policy of the institutes.
